# Long-Term Attrition in a 12-Month Adapted Physical Activity Program After Cardiac Rehabilitation: A Real-World Longitudinal Study

**DOI:** 10.3390/jcdd13090418

**Published:** 2026-08-27

**Authors:** Francesca Coppi, Gianluca Pagnoni, Aurora Vicenzi, Susan Darroudi, Gustavo Savino, Cecilia Zurlo, Laura Bernaroli, Francesco Marangi, Milena Nasi, Marcello Pinti, Alessio Baccarani, Anna Vittoria Mattioli, Francesco Fedele, Gilda Sandri

**Affiliations:** 1 National Institute for Cardiovascular Research (INRC), Via Irnerio 48, 40126 Bologna, Italy; 2 Department of Medical and Surgical Sciences for Children and Adults, University of Modena and Reggio Emilia, Via del Pozzo 71, 41124 Modena, Italy; 3 Cardiology Unit of Emergency Department, Guglielmo da Saliceto Hospital, 29121 Piacenza, Italy; 4 Department of Public Healthcare, Sports Medicine Service, Azienda USL of Modena, Via Rita Levi Montalcini 60-62, 41122 Modena, Italy; 5 Cardiology Division, Department of Biomedical Metabolic and Neural Sciences, University of Modena and Reggio Emilia, Via del Pozzo 71, 41124 Modena, Italy; 6 Department of Surgery, Medicine, Dentistry, and Morphological Sciences with Interest in Transplant, Oncology, and Regenerative Medicine, University of Modena and Reggio Emilia, 41125 Modena, Italy; 7 Department of Life Sciences, University of Modena and Reggio Emilia, Via G. Campi 287, 41125 Modena, Italy; 8 Division of Plastic and Reconstructive Surgery, Department of Medical and Surgical Sciences, Modena Policlinico Hospital, University of Modena and Reggio Emilia, 41124 Modena, Italy; alessio.baccarani@unimore.it; 9 Department of Quality of Life, University of Bologna-Alma Mater Studiorum, 40126 Bologna, Italy; 10 Department of Clinical, Internal, Anesthesiology and Cardiovascular Sciences, Sapienza University of Rome, Viale del Policlinico 155, 00161 Rome, Italy; 11 Rheumatology Unit, University of Modena and Reggio Emilia, 41125 Modena, Italy

**Keywords:** cardiac rehabilitation, Adapted Physical Activity (APA), attrition, retention, adherence, functional capacity, exercise training

## Abstract

**Background**: Exercise-based cardiac rehabilitation (CR) is a cornerstone of secondary cardiovascular prevention and improves functional capacity, quality of life, and clinical outcomes. However, maintaining long-term participation remains a major challenge in routine clinical practice. Adapted Physical Activity (APA) programs represent a community-based strategy to promote continued exercise after outpatient cardiac disease, although evidence regarding long-term retention and functional outcomes in real-world settings remains limited. The aim of this study was to evaluate attrition patterns during a 12-month APA follow-up pathway following cardiac rehabilitation and to explore longitudinal changes in functional outcomes among participants who remained under follow-up. **Methods**: This retrospective longitudinal observational study included 78 consecutive patients referred to an APA program following outpatient cardiac rehabilitation for myocardial infarction or other cardiovascular conditions. Assessments were performed at baseline (T0), 2 months (T1), 6 months (T2), and 12 months (T3). The primary outcome was attrition, operationally defined as failure to attend scheduled follow-up assessments. Secondary outcomes included walking speed during the 1 km Treadmill Walk Test, lower-limb functional performance, handgrip strength, balance, pain, and self-reported physical activity. Longitudinal changes were analyzed using linear mixed-effects models adjusted for age and sex. **Results**: Seventy-eight patients were enrolled (48 men, 29 women, one participant with missing sex data; mean age 64 ± 11 years). Follow-up attendance progressively declined from 81% at 2 months to 27% at 6 months and 18% at 12 months. Among participants with available follow-up data, significant improvements were observed in walking speed, lower-limb performance, balance, and motor pain during the early supervised phase of the program, with the most robust changes evident at the 2-month assessment (all *p* < 0.001). Although favorable values were also observed at later assessments, the marked loss to follow-up substantially limited interpretation of the 6- and 12-month estimates. Handgrip strength showed no significant longitudinal changes, while self-reported physical activity increased at 6 months but remained highly variable across participants. **Conclusions**: In this real-world APA follow-up pathway after cardiac rehabilitation, substantial attrition was observed, with fewer than one-fifth of participants attending follow-up assessments at 12 months. The most robust secondary finding was the short-term improvement in functional outcomes during the initial supervised phase. Long-term functional estimates should be considered exploratory because of the high attrition rate and potential survivor/selection bias. These findings highlight long-term retention as a key outcome for future community-based exercise programs and support the development of strategies aimed at improving sustained participation after cardiac rehabilitation.

## 1. Introduction

Cardiovascular diseases remain a leading cause of morbidity and mortality worldwide despite substantial advances in pharmacological and interventional therapies. Exercise-based cardiac rehabilitation (CR) is a cornerstone of secondary prevention and is strongly recommended by contemporary guidelines because of its beneficial effects on cardiovascular risk factor control, functional capacity, quality of life, and long-term clinical outcomes [1,2]. Modern rehabilitation models emphasize that the benefits of CR extend beyond the supervised exercise phase and depend on the adoption and maintenance of long-term healthy lifestyle behaviors and regular physical activity [3]. Despite robust evidence supporting CR, participation and adherence remain suboptimal in routine clinical practice. Enrollment rates are often low, and a substantial proportion of patients discontinue rehabilitation before completing the prescribed program [4,5,6]. Multiple barriers contribute to reduced participation and adherence, including transportation difficulties, geographic distance from rehabilitation facilities, work and family commitments, financial constraints, limited social support, and concerns regarding the safety or perceived necessity of exercise following a cardiovascular event [7,8,9]. Consequently, improving long-term engagement has become a major objective of contemporary cardiovascular prevention strategies [10].

Adapted Physical Activity (APA) programs provide individualized exercise interventions tailored to patients’ functional status and clinical characteristics and may represent a useful strategy to support long-term participation in exercise-based rehabilitation [11,12]. Compared with conventional hospital-based rehabilitation pathways, APA programs are frequently delivered in outpatient or community settings and may improve accessibility and continuity of care. While the beneficial effects of exercise training on functional outcomes are well established, evidence regarding long-term participation and retention in community-based APA programs remains limited [13,14].

Attrition has increasingly been recognized as a critical outcome in cardiac rehabilitation research because sustained participation is necessary to achieve and maintain the benefits associated with exercise training [15,16,17]. Previous studies have identified several factors associated with rehabilitation dropout, including younger age, female sex, socioeconomic disadvantage, psychological distress, and reduced referral or support pathways [18,19]. However, most available evidence derives from conventional cardiac rehabilitation settings, whereas longitudinal data describing retention and functional trajectories in APA-based programs remain scarce [20,21,22].

Despite the recognized role of Adapted Physical Activity (APA) programs in promoting long-term exercise participation after cardiac rehabilitation, evidence describing real-world retention patterns and their relationship with functional changes remains limited. Better understanding of attrition in routine clinical practice is essential for designing strategies aimed at improving long-term adherence and maximizing the benefits of exercise-based secondary prevention.

Because retention alone does not provide information on the potential functional trajectory of participants who remain engaged in the program, secondary functional outcomes were included to provide an exploratory description of changes occurring among individuals who continued attending follow-up assessments.

Therefore, the primary aim of the present study was to evaluate attrition patterns during a 12-month Adapted Physical Activity (APA) follow-up pathway delivered to patients with cardiovascular conditions referred for exercise-based cardiac rehabilitation. A secondary aim was to explore longitudinal changes in functional outcomes, including walking speed, lower-limb performance, balance, pain, and self-reported physical activity among participants attending follow-up assessments. We hypothesized that attrition would progressively increase over time and that the greatest functional improvements would occur during the early phases of supervised participation.

## 2. Methods

### 2.1. Study Design and Population

This retrospective longitudinal observational study included consecutive patients enrolled in a supervised outpatient Adapted Physical Activity (APA) program at the Sports Medicine Service of Azienda USL di Modena, Italy, between March 2021 and May 2025.

Eligible participants were adults (≥18 years) with cardiovascular disease who had completed a conventional outpatient cardiac rehabilitation program and were subsequently referred by their treating cardiologist to a long-term Adapted Physical Activity (APA) program as part of routine post-rehabilitation care. Referral was based on clinical stability and the need to continue supervised exercise following completion of the hospital-based cardiac rehabilitation pathway. The study population included patients with previous acute myocardial infarction (STEMI or NSTEMI), heart failure, valvular heart disease, atrial fibrillation, post-coronary artery bypass grafting (CABG), and other cardiovascular conditions requiring structured exercise intervention. All participants received medical clearance for exercise participation before enrollment.

Participants were referred by their treating cardiologists to the community-based Adapted Physical Activity (APA) program according to routine clinical practice. Referral occurred when continued supervised exercise was considered clinically appropriate following the acute management of the cardiovascular condition. Because the objective of the present study was to evaluate real-world participation and retention within the APA program, previous participation in hospital-based cardiac rehabilitation was not considered an exclusion criterion.

Exclusion criteria included unstable angina, decompensated heart failure, severe orthopedic or neurological conditions limiting exercise participation, and inability to provide informed consent.

The APA service receives referrals from a broad spectrum of cardiac rehabilitation indications, and the present study was intended to reflect routine clinical, practice rather than disease-specific, rehabilitation outcomes.

The study was conducted in accordance with the Declaration of Helsinki. The analyses were based exclusively on retrospectively collected and fully anonymized data generated during routine clinical practice. According to Italian national regulations governing retrospective observational studies based on anonymized data, formal approval by an Ethics Committee was not required.

### 2.2. Intervention

Adapted Physical Activity (APA) is a structured, non-healthcare exercise program designed for clinically stable individuals with chronic diseases, disabilities, or functional limitations. In Italy, APA programs are typically delivered in community settings as group-based exercise interventions and are intended to maintain and further improve the functional benefits achieved during the initial rehabilitation phase. Their primary objectives are to preserve mobility, functional autonomy, physical performance, and quality of life while preventing the progression of disability associated with chronic diseases.

The program is delivered by Adapted Physical Activity Kinesiologists (AMPA Kinesiologists), university-trained exercise professionals specialized in exercise prescription and supervision for individuals with chronic diseases and functional limitations, working in close collaboration with the referring cardiologists.

Participants were enrolled in a 12-month APA follow-up pathway, with an initial supervised phase consisting of two exercise sessions per week, each lasting approximately 60 min.

During the initial phase of the program (from baseline to the 2-month follow-up, T1), participants attended two supervised exercise sessions per week at the Sports Medicine Service of the Azienda USL of Modena. Following the T1 assessment, participants were referred to community-based APA facilities closer to their place of residence or, in a limited number of cases, were encouraged to continue the prescribed exercise program independently. Participants were subsequently invited to return to the Sports Medicine Service for follow-up functional assessments at 6 months (T2) and 12 months (T3).

Sessions were supervised by qualified exercise professionals operating within the Sports Medicine Service and were individualized according to each participant’s clinical condition and functional capacity. The exercise program included:

- warm-up activities (approximately 10 min);
- aerobic exercise (treadmill walking, cycle ergometer, or elliptical training) performed at moderate intensity corresponding to approximately 60–80% of peak heart rate (approximately 30 min);
- resistance and functional exercises using body weight, elastic bands, or light external loads (approximately 15 min);
- cool-down and stretching exercises (approximately 5 min).

Exercise intensity was prescribed according to the cardiologist’s exercise recommendation and the participant’s baseline functional capacity. Aerobic exercise was generally performed at moderate intensity (approximately 60–80% of peak heart rate) and monitored using heart rate, symptom evaluation, and the Borg Rating of Perceived Exertion scale. Exercise workload was progressively increased only when participants completed the prescribed training without adverse symptoms, according to individual clinical status, perceived exertion, and functional performance assessed during follow-up evaluations. No serious exercise-related adverse events were recorded during the supervised exercise sessions conducted at the Sports Medicine Service. Information regarding adverse events occurring after referral to community-based APA facilities or during independent exercise was not systematically collected.

Because exercise performed after referral to community APA facilities or during independent training was not supervised by the study center, attendance at individual exercise sessions after T1 was not systematically recorded.

### 2.3. Outcome Measures

Assessments were performed at baseline (T0), 2 months (T1), 6 months (T2), and 12 months (T3). The primary outcome was attrition, operationally defined as failure to attend the scheduled follow-up assessments (T1, T2, and T3). Within the present study, non-attendance at follow-up evaluations was considered indicative of withdrawal from the longitudinal APA follow-up pathway. Because participants continued the exercise program either in community-based APA facilities or independently after T1, attendance at every exercise session could not be systematically monitored. Consequently, follow-up attendance represented the predefined indicator used to assess continued participation in the long-term APA pathway. Although this definition does not allow direct quantification of attendance at every supervised exercise session, it reflects the predefined criterion routinely adopted within the regional APA pathway to identify participants who remained actively engaged in the longitudinal follow-up program.

Secondary outcomes included:

- 1 km Treadmill Walk Test (1 km TWT): Walking performance was assessed using the standardized 1 km Treadmill Walk Test, a perceptually regulated submaximal exercise test validated in patients with cardiovascular disease [23]. The test was performed on a motorized treadmill at 0% inclination according to the regional APA protocol. Participants initially walked at 2.0 km/h, and treadmill speed was progressively increased by 0.3 km/h every 30 s until a moderate exercise intensity corresponding to 11–13 on the Borg Rating of Perceived Exertion (6–20) scale was achieved. At this point, the timed 1 km test commenced. During the test, perceived exertion was reassessed every 2 min and treadmill speed was adjusted, when necessary, to maintain the target exercise intensity. Walking speed (m/min) was calculated by dividing the fixed distance (1000 m) by the completion time.
- 10 Sit to Stand Test: Time (seconds) to complete 10 repetitions of standing from a seated position, assessing lower-limb power.
- Handgrip Strength: Maximum grip force (kg) measured with a calibrated hydraulic dynamometer using the dominant upper limb. Three maximal trials were performed, and the highest value was retained for analysis.
- EQ Balance Score: A composite balance assessment (0–20 scale, lower scores indicate better balance).
- Visual Analog Scale (VAS) Pain: Self-reported motor pain and spine pain (0–100 scale, higher scores indicate worse pain).
- International Physical Activity Questionnaire (IPAQ): Self-reported physical activity (MET-min/week). The IPAQ was administered at baseline (T0) and during the 6- and 12-month follow-up visits (T2 and T3), but not at the 2-month assessment (T1), because T1 primarily focused on evaluating functional responses to the supervised exercise phase. Self-reported physical activity was assessed after transition to community-based or independent exercise to explore habitual activity beyond the supervised program.

### 2.4. Statistical Analysis

Continuous variables are summarized as mean ± standard deviation (SD) and categorical variables as count (percent). Within-patient change over time was modeled with linear mixed-effects models (random intercept per patient, restricted maximum likelihood). Visit was specified as a four-level categorical factor (T0, T1, T2, T3), with age and sex as fixed-effect covariates. Each contrast (T1 vs. T0, T2 vs. T0, T3 vs. T0) is reported with 95% confidence interval and two-sided *p*-value. Missing data were assumed missing at random under the mixed-effects framework. Given the substantial attrition observed during follow-up, the missing-at-random assumption may not have been fully satisfied; therefore, findings from later follow-up assessments should be interpreted cautiously and considered exploratory. Sensitivity analyses included paired within-patient Wilcoxon signed-rank tests at each follow-up and restriction to the 18 patients with confirmed myocardial infarction. Family-wise error across seven pre-specified outcomes was controlled with the Holm–Bonferroni procedure at α = 0.05.

## 3. Results

### 3.1. Cohort Characteristics

Seventy-eight patients were enrolled (48 men, 29 women, 1 missing; mean age 64 ± 11 years). Mean baseline body mass index was 29 ± 5 kg/m^2^ (*n* = 76); mean weight 84 ± 16 kg. Eighteen patients (23.1%) had documented acute myocardial infarction (STEMI or NSTEMI); the remainder had valvular disease, hypertensive heart disease, cardiomyopathy, atrial fibrillation, post-CABG status, or other cardiac indications. Baseline characteristics are reported in Table 1.

### 3.2. Attrition

**Figure 1:**
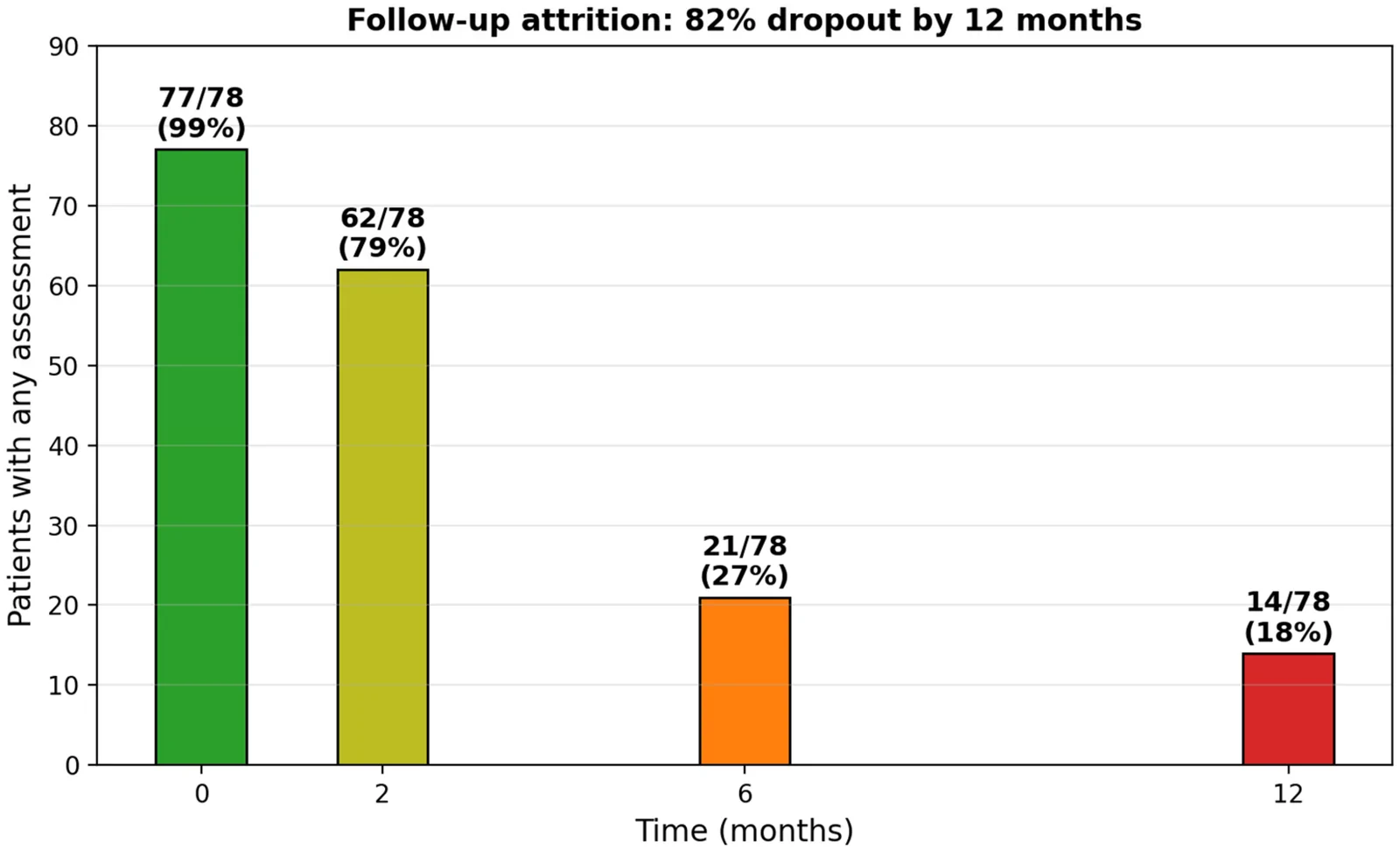
Participant retention during the 12-month adapted physical activity (APA) program, defined as attendance at each scheduled follow-up assessment.

Of 78 enrolled patients, 77 (99%) were assessed at baseline (T0), 63 (81%) at 2 months (T1), 21 (27%) at 6 months (T2), and 14 (18%) at 12 months (T3). Documented reasons for withdrawal included intercurrent illness (*n* = 6) and economic constraints (*n* = 1); reasons were not recorded for the remaining patients lost to follow-up (Figure 1). The attrition rate was 19% by 2 months, 73% by 6 months, and 82% by 12 months. These findings indicate substantial challenges in maintaining long-term participation in this real-world rehabilitation setting. Because reasons for withdrawal were unavailable for most participants, the determinants of attrition could not be explored further [24,25,26].

### 3.3. Walking Speed

**Figure 2:**
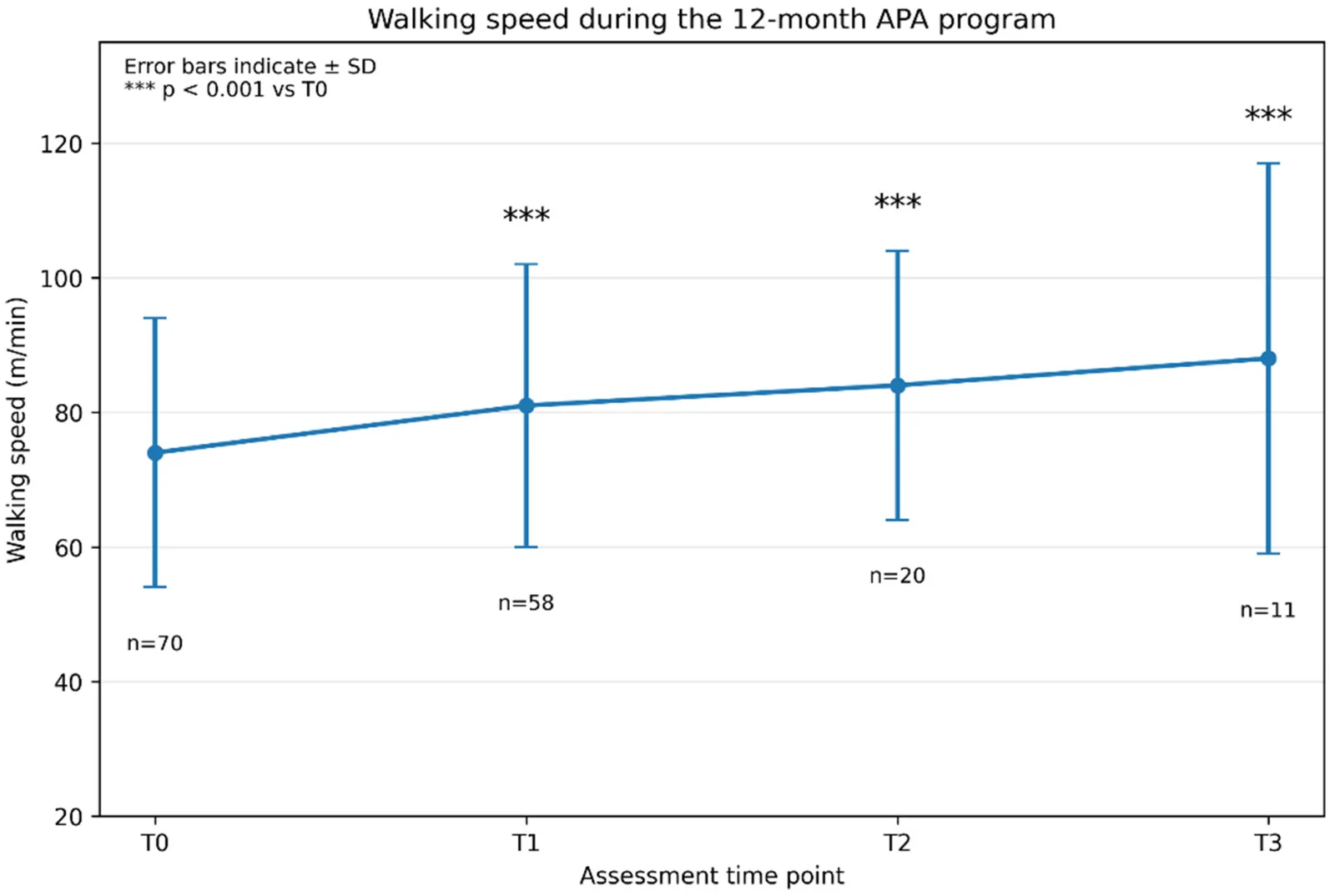
Individual trajectories of walking speed during the 12-month APA program. Blue lines represent individual participant trajectories, whereas blue circles indicate the cohort mean ± standard deviation (SD). *** *p* < 0.001 versus baseline (T0).

Walking speed was significantly higher than baseline at each available follow-up assessment. Descriptive values across study visits are reported in Table 2. In the mixed-effects model, all visit-specific contrasts versus baseline were statistically significant: β = 8.92 m/min (95% CI 6.75–11.09, *p* < 0.001) at T1 vs. T0; β = 9.46 m/min (95% CI 6.16–12.77, *p* < 0.001) at T2 vs. T0; and β = 12.61 m/min (95% CI 8.14–17.09, *p* < 0.001) at T3 vs. T0 (Table 3). Within-patient paired comparisons confirmed the effect (T0 → T1 mean Δ = 7.8 m/min, n = 57, Wilcoxon *p* < 0.001; T0 → T3 mean Δ = 13.1 m/min, n = 11, *p* = 0.004). Because the statistical analyses compared each follow-up assessment with baseline, rather than directly comparing consecutive follow-up visits, these findings should not be interpreted as evidence of progressive improvement from T1 to T3. However, given the marked reduction in sample size at later assessments, these estimates should be interpreted with caution because participants remaining under observation may not be representative of the original cohort [27,28,29]. Individual trajectories of walking speed across assessment time points are shown in Figure 2.

### 3.4. Strength, Balance and Physical Performance

Descriptive values for the 10 Sit-to-Stand Test across all visits are presented in Table 2. Mixed-effects analysis demonstrated significant improvements at T1 (β = −3.31 s, 95% CI −5.11 to −1.52, *p* < 0.001) and T2 (β = −3.59 s, 95% CI −6.34 to −0.83, *p* = 0.011), but the T3 contrast was not significant (β = +1.41 s, *p* = 0.41) (Table 3). These findings indicate better lower-limb functional performance relative to baseline at T1 and T2, whereas no significant difference from baseline was observed at T3. Given the limited number of participants available at later assessments, these findings should be interpreted cautiously [30,31,32].

Handgrip strength remained essentially unchanged throughout follow-up, with no statistically significant differences observed across study visits (Table 3). Corresponding values are presented in Table 2. These findings suggest that changes observed in other functional outcomes were not accompanied by detectable changes in handgrip strength within the study population [33,34].

The EQ balance score was significantly lower than baseline at T1 (β = −3.96, 95% CI −5.09 to −2.82, *p* < 0.001) and T2 (β = −2.29, 95% CI −4.01 to −0.58, *p* = 0.009), whereas no significant difference from baseline was observed at T3 (β = +0.45, *p* = 0.71) (Table 3). Thus, differences from baseline were evident at the earlier follow-up assessments but were not detectable at 12 months. Given the progressively smaller number of observations, the later estimates remain exploratory [35,36].

### 3.5. Pain and Self-Reported Physical Activity

Descriptive motor pain values are presented in Table 2, whereas model estimates are reported in Table 3. Motor pain was significantly lower than baseline at T1 (β = −14.51, 95% CI −22.41 to −6.62, *p* < 0.001). Although mean pain scores were also numerically lower than baseline at T2 and T3, these contrasts were not statistically significant. Therefore, the later findings should not be interpreted as evidence of maintenance or further improvement beyond T1, particularly given the substantial reduction in the number of participants available for assessment [37,38].

Descriptive spine pain values are reported in Table 2, whereas model estimates are presented in Table 3. Spine pain was significantly lower than baseline at T1, whereas no significant differences from baseline were observed at T2 or T3 [39,40].

**Figure 3:**
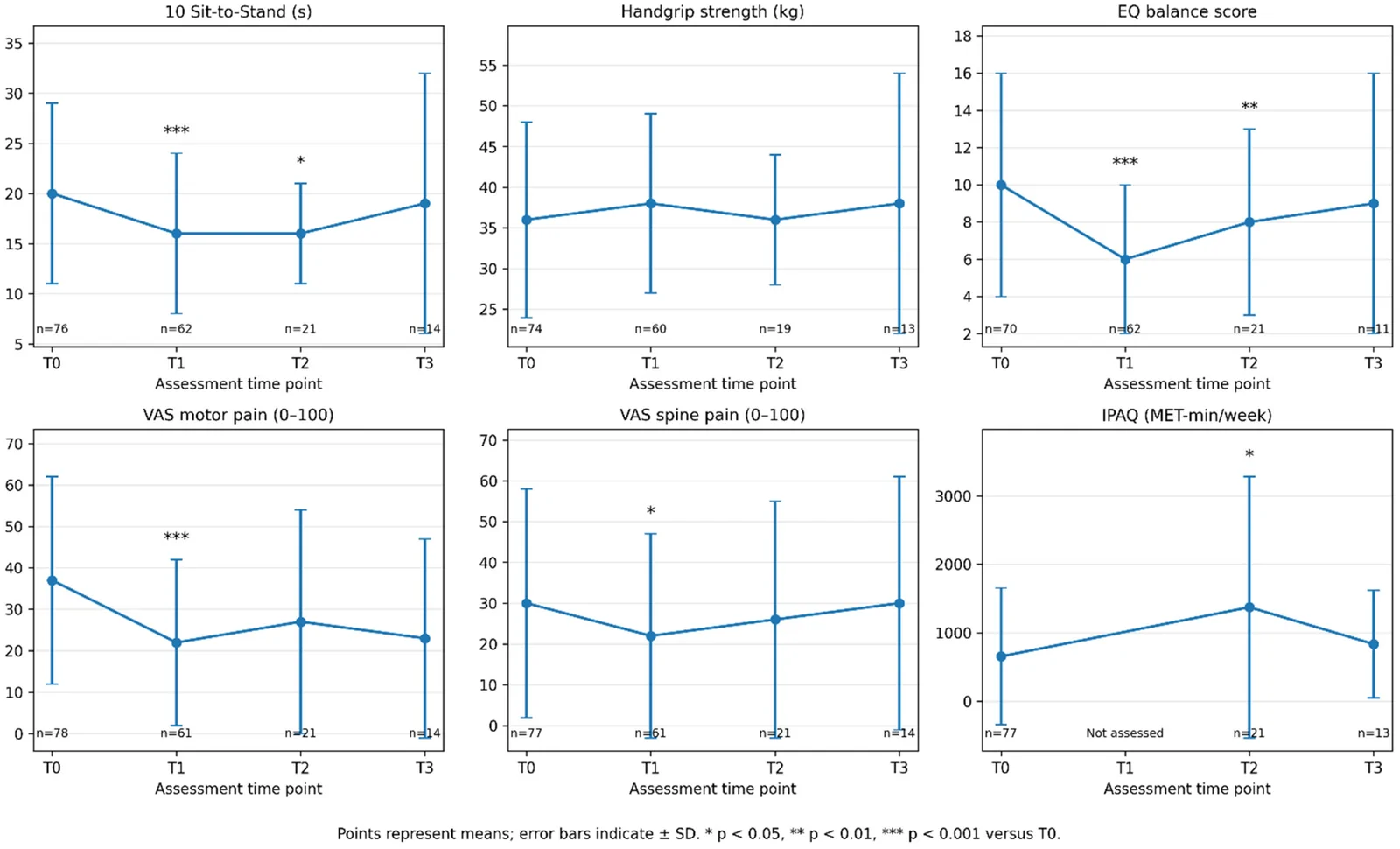
Longitudinal changes in secondary outcomes during the 12-month APA program. Data are presented as mean ± standard deviation (SD). Statistical significance versus baseline (T0) is indicated as * *p* < 0.05, ** *p* < 0.01 and *** *p* < 0.001.

Self-reported physical activity appeared to increase at the 6-month assessment (Table 3). However, the substantial inter-individual variability and the limited number of participants available at later follow-up visits suggest that these findings should be interpreted as exploratory. Corresponding IPAQ values are reported in Table 2 [41,42]. Longitudinal changes in the secondary functional outcomes are summarized in Figure 3.

### 3.6. Sensitivity Analyses

In the MI-only subgroup (*n* = 18), the direction of effect for walking speed was preserved with effect sizes larger than in the full cohort: walking speed at T1 vs. T0 β = 13.34 m/min (95% CI 9.08–17.61, *p* < 0.001), at T3 vs. T0 β = 22.63 m/min (95% CI 14.76–30.50, *p* < 0.001). EQ balance at T1 also remained highly significant (β = −4.88, *p* < 0.001), while 10 Sit-to-Stand contrasts in the MI subgroup lost significance owing to limited statistical power (Table 4).

Paired Wilcoxon signed-rank tests confirmed the direction and significance of the mixed-effects model contrasts at T1 across all primary and most secondary outcomes. The T0 → T1 paired changes were highly significant for walking speed (Δ = 7.82, *n* = 57, *p* < 0.001), 10 Sit-to-Stand (Δ = −3.06, *n* = 61, *p* = 0.002), EQ balance (Δ = −3.70, *n* = 56, *p* < 0.001), VAS motor pain (Δ = −14.28, *n* = 61, *p* = 0.002), and VAS spine pain (Δ = −8.25, *n* = 60, *p* = 0.052). The 2-month change emerges as the most robust finding across every sensitivity specification, non-parametric, MI-restricted, and multiplicity-adjusted, and constitutes the primary defensible result of the study [43,44].

## 4. Discussion

This real-world longitudinal study evaluated attrition and exploratory functional changes among patients participating in a 12-month Adapted Physical Activity (APA) program following outpatient cardiac rehabilitation. The principal finding was the substantial decline in participant retention over time, with follow-up attendance decreasing from 81% at 2 months to only 18% at 12 months. Among participants who remained under follow-up, favorable short-term improvements were observed in several functional outcomes, particularly during the first two months of supervised exercise. However, because of the marked loss to follow-up at later time points, these findings should be considered exploratory and interpreted with caution, as they may reflect survivor and selection bias rather than the long-term effectiveness of the APA program. Therefore, the most robust finding of the present study is the combination of early functional improvement during the supervised phase, together with the progressive decline in long-term retention, highlighting retention itself as a critical outcome for future community-based cardiac rehabilitation programs.

Long-term adherence is a fundamental component of successful cardiac rehabilitation because the benefits of exercise-based interventions depend on sustained participation rather than short-term engagement alone [44,45]. This concept is consistent with previous evidence indicating that physical activity must be regular and sustained over time to confer the greatest cardiovascular protection and long-term health benefits [46].

Despite the well-established efficacy of cardiac rehabilitation, maintaining long-term attendance remains challenging across different healthcare systems and rehabilitation models [46,47,48,49]. However, our findings are broadly consistent with reports from real-world rehabilitation settings, particularly community-based and maintenance-phase programs, where progressive disengagement over time remains a major obstacle to effective secondary prevention [49,50,51,52]. Several factors may contribute to reduced long-term participation in exercise-based rehabilitation, including transportation difficulties, competing work and family responsibilities, financial constraints, limited social support, and concerns regarding exercise safety or necessity after a cardiovascular event [53,54,55,56,57]. Unfortunately, reasons for withdrawal were available for only a small proportion of participants in the present study, preventing formal analyses of determinants of attrition. Nevertheless, the magnitude of follow-up loss observed in our cohort highlights the importance of developing strategies specifically aimed at improving long-term retention. Home-based rehabilitation, telehealth-supported exercise programs, hybrid care models, behavioral interventions, and targeted socioeconomic support have all been proposed as potential approaches to improve adherence and continuity of participation [58,59,60,61].

### 4.1. Functional Outcomes

Among participants who continued attending follow-up assessments, walking speed was significantly higher than baseline at each available follow-up assessment. However, because the statistical analyses compared each follow-up visit with T0 and did not include direct comparisons between consecutive follow-up time points, these findings do not demonstrate progressive improvement beyond the initial supervised phase. Moreover, the marked attrition after T1 substantially limits interpretation of the later estimates. Accordingly, the T2 and T3 findings should be regarded as exploratory evidence that walking speed remained different from baseline among participants who returned for assessment, rather than as evidence of continued improvement over time. These findings are consistent with previous evidence demonstrating favorable effects of exercise-based rehabilitation on functional capacity and exercise performance among patients with cardiovascular disease.

Similarly, lower-limb functional performance and balance were significantly better than baseline at T1 and T2, whereas no significant differences from baseline were observed at T3. These findings are consistent with physiological adaptations commonly associated with regular supervised exercise training, including improvements in neuromuscular coordination, mobility and exercise tolerance [30,31]. In contrast, handgrip strength remained relatively stable throughout follow-up, whereas differences from baseline were more evident in mobility-related and lower-limb functional domains than in upper-limb strength measures [32,33]. The absence of meaningful changes in handgrip performance is not unexpected, given that the intervention primarily emphasized aerobic exercise and general functional conditioning rather than targeted strength training.

Pain-related outcomes showed a somewhat different pattern. Motor pain was significantly lower than baseline at T1, while mean values remained numerically lower at later assessments without statistically significant differences from baseline. Spine pain was also lower than baseline at T1, whereas no significant differences from baseline were detected at T2 or T3 [36,37]. These findings therefore support an early association between supervised participation and pain-related outcomes but do not establish persistence or progression of these changes over time. Moreover, causal interpretations cannot be made because of the observational study design and the absence of a control group.

Self-reported physical activity increased at 6 months but was characterized by considerable inter-individual variability and a limited sample size at later assessments. Consequently, these findings should be considered exploratory and interpreted cautiously [38,39,40,41].

### 4.2. Clinical Implications

The present findings provide pragmatic information on which components of the current Adapted Physical Activity (APA) pathway appeared feasible in routine practice and which components may require refinement. The initial two-month phase, characterized by twice-weekly supervised exercise at the Sports Medicine Service and scheduled functional reassessment, achieved relatively high follow-up attendance, with 81% of participants returning for the T1 assessment. No serious exercise-related adverse events were recorded during supervised sessions. These observations suggest that the structured, supervised, and closely monitored early phase was feasible within routine clinical practice, although the present study was not designed to establish its effectiveness.

The main implementation challenge emerged during longer-term follow-up. After T1, participants transitioned from center-based supervision to community-based APA facilities closer to their place of residence or, in a limited number of cases, to independent exercise, while being invited to return to the Sports Medicine Service for assessments at T2 and T3. Follow-up attendance decreased substantially from 81% at T1 to 27% at T2 and 18% at T3. Importantly, this decline cannot be attributed to the transition itself, because exercise session attendance after T1 and reasons for non-attendance at follow-up assessments were not systematically recorded. Nevertheless, the temporal pattern identifies the transition from center-based supervision to decentralized exercise as a potentially critical implementation point that warrants prospective evaluation. This is consistent with previous evidence showing that maintaining long-term participation remains challenging across rehabilitation models and that sustained physical activity after completion of structured cardiac rehabilitation represents an important challenge in secondary prevention.

From an implementation perspective, the main area requiring refinement is therefore the continuity of the pathway after transition from center-based supervision. Potential revisions could include a structured handover from the Sports Medicine Service to the designated community APA provider, confirmation that the participant has successfully initiated the community-based program, predefined communication between referring clinicians and exercise professionals, systematic recording of exercise attendance and reasons for discontinuation, and scheduled reminders before T2 and T3 follow-up assessments. These measures would also allow future evaluations to distinguish loss to clinical follow-up from actual discontinuation of exercise participation. Flexible, hybrid, or telehealth-supported follow-up could additionally be considered for participants facing geographic, organizational, or personal barriers [42,43].

Telehealth-supported and hybrid rehabilitation approaches may be particularly useful when geographic, organizational, or personal barriers limit attendance at center-based follow-up, while preserving periodic contact with the referring clinical team [62]. These approaches should be regarded as potential implementation strategies, rather than interventions demonstrated to be effective by the present study, and should be prospectively evaluated for their ability to improve retention. More broadly, the implementation of long-term community-based exercise pathways should remain individualized according to patients’ clinical characteristics and exercise tolerance and embedded within an appropriate cardiovascular safety framework [63,64,65].

Overall, the substantial attrition observed in this cohort indicates that retention should be considered a key feasibility and implementation outcome when evaluating long-term community-based APA pathways. Functional outcomes collected at later follow-up visits become increasingly difficult to interpret when only a small proportion of the original cohort remains under observation. Future prospective studies should therefore assess not only functional outcomes, but also implementation outcomes, including retention, actual exercise attendance, reasons for discontinuation, successful transition to community-based exercise, and barriers to follow-up. Such information would help determine which components of the APA pathway are feasible in routine practice, which require modification, and whether specific implementation strategies can support sustained engagement over time.

### 4.3. Strengths and Limitations

Several strengths of this study should be acknowledged. First, the study reflects routine clinical practice within a real-world outpatient APA program, providing information that may complement evidence derived from highly controlled clinical trials. Second, participants were followed for up to 12 months, allowing evaluation of both short-term and longer-term participation patterns. Third, longitudinal analyses were performed using mixed-effects models that allowed inclusion of all available observations while accounting for repeated measurements within individuals.

The study also has important limitations. The retrospective observational design precludes causal inference regarding the effects of the intervention. The study population was heterogeneous and included patients referred for different cardiovascular rehabilitation indications, which may limit direct generalizability to specific diagnostic groups, particularly because comorbidities such as obesity may modify clinical characteristics, prognosis, and risk stratification in selected conditions such as pulmonary arterial hypertension [66]. More detailed echocardiographic phenotyping was unavailable; future studies should incorporate a multiparametric assessment of right ventricular function, including TAPSE, the more reproducible TDI S′, and, in patients with suspected pulmonary hypertension, the TAPSE/sPAP ratio [67,68]. Background pharmacological therapy was also not systematically analyzed and may represent a potential confounder, particularly after PCI, given the effects of agents such as empagliflozin on inflammatory and oxidative stress pathways [69]. Most importantly, substantial follow-up attrition introduced the possibility of selection bias and reduced the precision of long-term outcome estimates. Participants who remained under follow-up at the later assessment time points may have represented a more motivated, healthier, or functionally better-performing subgroup of the original cohort. Consequently, the functional differences observed relative to baseline at later follow-up assessments may have been influenced by survivor and selection bias and should therefore be interpreted as exploratory rather than as evidence of sustained or progressive improvement.

Because attendance at assessment visits was used to define retention, loss to follow-up may not necessarily reflect complete discontinuation of exercise participation. Moreover, because attendance at individual exercise sessions after referral to community-based Adapted Physical Activity (APA) facilities or independent exercise was not systematically recorded, actual adherence to the prescribed exercise program could not be directly quantified. Consequently, follow-up attendance should be interpreted as a pragmatic indicator of continued engagement within the APA pathway rather than as a direct measure of long-term exercise adherence. Furthermore, reasons for withdrawal were not systematically recorded for most participants, preventing a detailed investigation of predictors of attrition. Finally, the single-center design may limit the generalizability of the findings to other healthcare settings and rehabilitation models.

Future prospective studies should prioritize the identification of predictors of attrition and evaluate interventions specifically designed to improve long-term participation in exercise-based rehabilitation. Better understanding of the demographic, clinical, psychological, and socioeconomic factors associated with disengagement may help develop targeted strategies to enhance retention and maximize the long-term benefits of cardiac rehabilitation programs.

## 5. Conclusions

In this real-world cohort of patients participating in a 12-month Adapted Physical Activity (APA) program, substantial attrition was observed throughout follow-up, with participant retention progressively declining over time. Among participants who remained under observation, favorable short-term improvements were observed in walking speed and selected functional outcomes during the initial supervised phase of the program. However, because of the marked loss to follow-up at later assessment time points, these differences from baseline at later follow-up assessments should be considered exploratory and interpreted with caution, as they may have been influenced by survivor and selection bias. Overall, these findings support the concept that long-term retention should be regarded as a key quality indicator of community-based Adapted Physical Activity (APA) programs. Future prospective studies should evaluate implementation strategies aimed at strengthening the transition from center-based supervision to community-based or independent exercise, including structured handover procedures, systematic attendance monitoring, and flexible follow-up models. Such approaches should be formally evaluated for their ability to improve long-term retention within APA pathways.

## Figures and Tables

**Table 1:** Baseline characteristics of the study cohort (*n* = 78).

Characteristic	Value	N
Age, years	64 ± 11	78
Sex, Male	48 (62%)	78
Sex, Female	29 (37%)	78
Weight, kg	84 ± 16	77
BMI, kg/m^2^	29 ± 5	76
Confirmed STEMI/NSTEMI	18 (23%)	78
Other cardiac indication	60 (77%)	78
1 km TWT walking speed, m/min	74 ± 20	70
10 Sit-to-Stand, s	20 ± 9	76
Handgrip, kg	36 ± 12	74
VAS motor pain, 0–100	37 ± 25	78
VAS spine pain, 0–100	30 ± 28	77

*Data presented as mean ± SD or number (percent). BMI = body mass index; TWT = Treadmill Walk Test; VAS = Visual Analog Scale.*

**Table 2:** Outcomes across visits over 12 months.

Outcome	T0 (Baseline)	T1 (2 Months)	T2 (6 Months)	T3 (12 Months)
1 km TWT speed, m/min	74 ± 20 (70)	81 ± 21 (58)	84 ± 20 (20)	88 ± 29 (11)
10 Sit-to-Stand, s	20 ± 9 (76)	16 ± 8 (62)	16 ± 5 (21)	19 ± 13 (14)
Handgrip, kg	36 ± 12 (74)	38 ± 11 (60)	36 ± 8 (19)	38 ± 16 (13)
EQ balance score	10 ± 6 (70)	6 ± 4 (62)	8 ± 5 (21)	9 ± 7 (11)
VAS motor pain, 0–100	37 ± 25 (78)	22 ± 20 (61)	27 ± 27 (21)	23 ± 24 (14)
VAS spine pain, 0–100	30 ± 28 (77)	22 ± 25 (61)	26 ± 29 (21)	30 ± 31 (14)
IPAQ, MET-min/week	656 ± 995 (77)	N.A.	1373 ± 1907 (21)	835 ± 786 (13)

*Data presented as mean ± SD N.A. = not assessable (variable not recorded at this visit in the source database). TWT = Treadmill Walking Test; VAS = Visual Analog Scale (0–100); IPAQ = International Physical Activity Questionnaire. Lower values indicate improvement for 10 Sit-to-Stand, EQ balance, and VAS.*

**Table 3:** Mixed-effects model results: visit-specific contrasts vs. T0, adjusted for age and sex.

Outcome	T1 (β and 95% CI)	T2 (β and 95% CI)	T3 (β and 95% CI)
1 km TWT speed (m/min)	8.92 (6.75, 11.09)	9.46 (6.16, 12.77)	12.61 (8.14, 17.09)
10 Sit-to-Stand (s)	−3.31 (−5.11, −1.52)	−3.59 (−6.34, −0.83)	1.41 (−1.94, 4.77)
EQ balance score	−3.96 (−5.09, −2.82)	−2.29 (−4.01, −0.58)	0.45 (−1.88, 2.77)
Hand grip (kg)	0.97 (−0.88, 2.83)	0.21 (−2.74, 3.16)	−1.65 (−5.19, 1.89)
VAS motor pain	−14.51 (−22.41, −6.62)	−10.05 (−21.40, 1.31)	−13.38 (−26.79, 0.03)
VAS spine pain	−7.02 (−13.20, −0.85)	0.16 (−9.26, 9.59)	−4.06 (−15.49, 7.37)
IPAQ (MET-min/wk)	-	716.7 (131.3, 1302.1)	179.4 (−533.6, 892.4)

*β represents the model-estimated mean difference from baseline at the indicated visit, adjusted for age and sex. Negative values for 10 Sit-to-Stand, EQ balance, and VAS pain scales indicate improvement. p < 0.05, p < 0.01, p < 0.001.*

**Table 4:** MI-only subgroup (*n* = 18)—mixed-effects, adjusted for age and sex.

Outcome	T1 (β and 95% CI)	T2 (β and 95% CI)	T3 (β and 95% CI)
1 km TWT speed (m/min)	13.34 (9.08, 17.61)	21.20 (13.34, 29.06)	22.63 (14.76, 30.50)
10 Sit-to-Stand (s)	−2.42 (−8.04, 3.21)	N.A.	−5.2 (−16.16, 5.76)
EQ balance score	−4.88 (−7.34, −2.42)	N.A.	−0.76 (−5.23, 3.71)

*Effect directions and significance are preserved for walking speed at T1; the magnitude of the speed effect is larger than in the full cohort. Secondary outcomes lose significance owing to limited power at late visits in this subgroup. p < 0.001.*

## Data Availability

The data presented in this study are available on request from the corresponding author.

## References

[1] Anderson L., Oldridge N., Thompson D.R., Zwisler A.D., Rees K., Martin N., Taylor R.S. (2016). Exercise-Based Cardiac Rehabilitation for Coronary Heart Disease: Cochrane Systematic Review and Meta-Analysis. J. Am. Coll. Cardiol..

[2] Taylor R.S., Long L., Mordi I.R., Madsen M.T., Davies E.J., Dalal H., Rees K., Singh S.J., Gluud C., Zwisler A.D. (2019). Exercise-Based Rehabilitation for Heart Failure: Cochrane Systematic Review, Meta-Analysis, and Trial Sequential Analysis. JACC Heart Fail..

[3] Khadanga S., Savage P., Keteyian S., Yant B., Gaalema D., Ades P. (2024). Cardiac rehabilitation: The gateway for secondary prevention. Heart.

[4] Ruano-Ravina A., Pena-Gil C., Abu-Assi E., Raposeiras S., van ‘t Hof A., Meindersma E., Bossano Prescott E.I., González-Juanatey J.R. (2016). Participation and adherence to cardiac rehabilitation programs. A systematic review. Int. J. Cardiol..

[5] Ades P.A., Keteyian S.J., Wright J.S., Hamm L.F., Lui K., Newlin K., Shepard D.S., Thomas R.J. (2017). Increasing Cardiac Rehabilitation Participation From 20% to 70%: A Road Map From the Million Hearts Cardiac Rehabilitation Collaborative. Mayo Clin. Proc..

[6] Ögmundsdottir Michelsen H., Sjölin I., Schlyter M., Hagström E., Kiessling A., Henriksson P., Held C., Hag E., Nilsson L., Bäck M. (2020). Cardiac rehabilitation after acute myocardial infarction in Sweden—Evaluation of programme characteristics and adherence to European guidelines: The Perfect Cardiac Rehabilitation (Perfect-CR) study. Eur. J. Prev. Cardiol..

[7] Pagnoni G., Vicenzi A., Darroudi S., Maini A., Sbarra F., Marangi F., Loffi M., Nasi M., Pinti M., Selleri V. (2025). Adherence to Exercise and Functional Rehabilitation Programs in Patients with Cardiovascular Diseases: Barriers and Strategies. J. Cardiovasc. Dev. Dis..

[8] Serves N., Pazart L., Gabriel D., Mourot L., Ecarnot F. (2023). Adherence to rehabilitation and home exercise after myocardial infarction: A qualitative study of expectations, barriers and drivers. BMC Sports Sci. Med. Rehabil..

[9] Zhao X., Zhang S., Zhang F., Wu X., Zhang Z., Liu Y., Jiang J., Li Z., Li S., Zheng S. (2025). Barriers and Facilitators to Adherence to Exercise-Based Cardiac Rehabilitation Among Coronary Artery Disease Patients: A Scoping Review. J. Multidiscip. Healthc..

[10] Visseren F.L.J., Mach F., Smulders Y.M., Carballo D., Koskinas K.C., Bäck M., Benetos A., Biffi A., Boavida J.M., Capodanno D. (2021). 2021 ESC Guidelines on cardiovascular disease prevention in clinical practice. Eur. Heart J..

[11] Greco G., Fischetti F. (2025). Adapted Exercise and Adapted Sport as Rights of Health Citizenship in Italy: A Legal–Policy Rationale and Framework for Inclusion in the Livelli Essenziali di Assistenza (LEA) and the Role of the Chinesiologo. Societies.

[12] Tonet E., Sabato F., Canovi L., Guidi Colombi G., Campo G., Guardigli G., Perone F. (2025). Physical activity and cardiac rehabilitation after myocardial infarction: The risk of obtaining large benefits. Panminerva Med..

[13] Cutore L., Faro D.C., Raffo C., Di Leo G., Sangiorgio G., Capodanno D. (2026). Post-acute coronary syndrome cardiovascular rehabilitation: Insights into endpoints, biomarkers and clinical practice. Heart.

[14] Coull A., Pugh G. (2021). Maintaining physical activity following myocardial infarction: A qualitative study. BMC Cardiovasc. Disord..

[15] Sommer C.G., Jørgensen L.B., Blume B., Møller T., Skou S.T., Harrison A., Tang L.H. (2022). Dropout during a 12-week transitional exercise-based cardiac rehabilitation programme: A mixed-methods prospective cohort study. Eur. J. Cardiovasc. Nurs..

[16] Li P., Zhang W., Wu B. (2024). Adherence to Cardiac Rehabilitation in Patients with Acute Myocardial Infarction After PCI: A Scoping Review. J. Multidiscip. Healthc..

[17] Iyngkaran P., Appuhamilage P.Y., Patabandige G., Sarathchandra Peru Kandage P.S., Usmani W., Hanna F. (2024). Barriers to Cardiac Rehabilitation among Patients Diagnosed with Cardiovascular Diseases-A Scoping Review. Int. J. Environ. Res. Public Health.

[18] Gaalema D.E., Savage P.D., Rengo J.L., Cutler A.Y., Elliott R.J., Priest J.S., Higgins S.T., Ades P.A. (2017). Patient Characteristics Predictive of Cardiac Rehabilitation Adherence. J. Cardiopulm. Rehabil. Prev..

[19] Forsyth F., Deaton C. (2021). Women and cardiac rehabilitation: Moving beyond barriers to solutions?. Eur. J. Prev. Cardiol..

[20] McFeely A.E., Bittner V.A. (2025). Sex and Gender-Based Differences in Outcomes of Cardiac Rehabilitation Following Acute Myocardial Infarction. Curr. Atheroscler. Rep..

[21] Mandic S., Body D., Barclay L., Walker R., Nye E.R., Grace S.L., Williams M.J. (2015). Community-Based Cardiac Rehabilitation Maintenance Programs: Use and Effects. Heart Lung Circ..

[22] Hardcastle S.J., McNamara K., Tritton L. (2015). Using Visual Methods to Understand Physical Activity Maintenance following Cardiac Rehabilitation. PLoS ONE.

[23] Mazzoni G., Chiaranda G., Myers J., Sassone B., Pasanisi G., Mandini S., Volpato S., Conconi F., Grazzi G. (2018). 500-meter and 1000-meter moderate walks equally assess cardiorespiratory fitness in male outpatients with cardiovascular diseases. J. Sports Med. Phys. Fit..

[24] Ozemek C., Squires R.W. (2021). Enrollment and Adherence to Early Outpatient and Maintenance Cardiac Rehabilitation Programs. J. Cardiopulm. Rehabil. Prev..

[25] Beleigoli A., Dafny H.A., Pinero de Plaza M.A., Hutchinson C., Marin T., Ramos J.S., Suebkinorn O., Gebremichael L.G., Bulamu N.B., Keech W. (2024). Clinical effectiveness of cardiac rehabilitation and barriers to completion in patients of low socioeconomic status in rural areas: A mixed-methods study. Clin. Rehabil..

[26] Lemos Pires M., Borges M., De Sa G., Novakovic M., Alves Da Silva P., Cunha N., Ricardo I., Pinto F.J., Pinto R., Melo X. (2024). Long-term attendance and dropout rates in a phase 3 cardiovascular rehabilitation program: Differences between men and women. Eur. J. Prev. Cardiol..

[27] Santaularia N., Caminal J., Arnau A., Perramon M., Montesinos J., Abenoza Guardiola M., Jaarsma T. (2017). The efficacy of a supervised exercise training programme on readmission rates in patients with myocardial ischemia: Results from a randomised controlled trial. Eur. J. Cardiovasc. Nurs..

[28] Popiolek-Kalisz J., Rabiej-Krzys P. (2026). The role of sex and age in response to early post-myocardial infarction cardiac rehabilitation. Curr. Probl. Cardiol..

[29] Hammer A., Sinkovec H., Todorovic M., Katsch F., Gall W., Duftschmid G., Heinze G., Niessner A., Sulzgruber P. (2024). Adherence to Secondary Prevention Measures after Acute Myocardial Infarction and Its Impact on Patient Outcome-A Nationwide Perspective. J. Clin. Med..

[30] Taylor J.L., Holland D.J., Keating S.E., Bonikowske A.R., Coombes J.S. (2021). Adherence to High-Intensity Interval Training in Cardiac Rehabilitation: A REVIEW AND RECOMMENDATIONS. J. Cardiopulm. Rehabil. Prev..

[31] Xia C., Zheng Y., Ji L., Liu H. (2024). Comparative effectiveness of different interventions on adherence to exercise-based CR among patients after percutaneous coronary intervention: A network meta-analysis of randomized controlled trials. BMC Nurs..

[32] Bracewell N.J., Plasschaert J., Conti C.R., Keeley E.C., Conti J.B. (2022). Cardiac rehabilitation: Effective yet underutilized in patients with cardiovascular disease. Clin. Cardiol..

[33] Ullah M., Rahman M.T., Islam A.M., Majumder A. (2023). Cardiac Rehabilitation in Coronary Artery Disease: Improving Outcomes and Adherence. Cardiovasc. J..

[34] Kodeboina M., Piayda K., Jenniskens I., Vyas P., Chen S., Pesigan R.J., Ferko N., Patel B.P., Dobrin A., Habib J. (2023). Challenges and Burdens in the Coronary Artery Disease Care Pathway for Patients Undergoing Percutaneous Coronary Intervention: A Contemporary Narrative Review. Int. J. Environ. Res. Public Health.

[35] Lesage-Moussavou-Nzamba M., Houle J., Trudeau F. (2020). Participants’ Perspectives of a Primary Exercise-Based Prevention Program for Cardiac Patients: A Prepost Intervention Qualitative Case Study. Rehabil. Res. Pract..

[36] Kim H., Yun R.Y., Kim C., Shin Y.I., Bang H.J., Uhm K.E., Kim S.H., Bae J.W., Lee K., Kim Y. (2026). Trends in Cardiac Rehabilitation Participation in Patients With Acute Myocardial Infarction: A 5-Year Nationwide Study in Korea. J. Korean Med. Sci..

[37] Chindhy S., Taub P.R., Lavie C.J., Shen J. (2020). Current challenges in cardiac rehabilitation: Strategies to overcome social factors and attendance barriers. Expert Rev. Cardiovasc. Ther..

[38] Dhaliwal K.K., King-Shier K., Manns B.J., Hemmelgarn B.R., Stone J.A., Campbell D.J. (2017). Exploring the impact of financial barriers on secondary prevention of heart disease. BMC Cardiovasc. Disord..

[39] McHale S., Astin F., Neubeck L., Dawkes S., Hanson C.L. (2020). A systematic review and thematic synthesis exploring how a previous experience of physical activity influences engagement with cardiac rehabilitation. Eur. J. Cardiovasc. Nurs..

[40] McCleary N., Ivers N.M., Schwalm J.D., Witteman H.O., Taljaard M., Desveaux L., Bouck Z., Grace S.L., Natarajan M.K., Grimshaw J.M. (2020). Interventions supporting cardiac rehabilitation completion: Process evaluation investigating theory-based mechanisms of action. Health Psychol..

[41] Giannoccaro J.D., Aggarwal S., Grace S.L., Campbell T.S., Hauer T., Arena R., Rouleau C.R. (2020). Factors Associated With Attendance at a 1-yr Post-Cardiac Rehabilitation Risk Factor Check. J. Cardiopulm. Rehabil. Prev..

[42] Pirruccello J.P., Traynor K., Aragam K.G. (2017). “Road Map” to Improving Enrollment in Cardiac Rehabilitation: Identifying Barriers and Evaluating Alternatives. J. Am. Heart Assoc..

[43] Crawshaw J., Corovic M., Ajlan M., Mosleh K.D., Natarajan M., Schwalm J.D. (2025). Strengthening Transitions in Care for Patients with ST-Elevation Myocardial Infarction: A Theory-Based Qualitative Study. CJC Open.

[44] Aktaa S., Gencer B., Arbelo E., Davos C.H., Désormais I., Hollander M., Abreu A., Ambrosetti M., Bäck M., Carballo D. (2022). European Society of Cardiology Quality Indicators for Cardiovascular Disease Prevention: Developed by the Working Group for Cardiovascular Disease Prevention Quality Indicators in collaboration with the European Association for Preventive Cardiology of the European Society of Cardiology. Eur. J. Prev. Cardiol..

[45] Mattioli A.V., Coppi F., Migaldi M., Farinetti A. (2018). Physical activity in premenopausal women with asymptomatic peripheral arterial disease. J. Cardiovasc. Med..

[46] Foster E.J., Munoz S.A., Crabtree D., Leslie S.J., Gorely T. (2021). Barriers and facilitators to participating in cardiac rehabilitation and physical activity in a remote and rural population: A cross-sectional survey. Cardiol. J..

[47] Bäck M., Leosdottir M., James S., Hagström E. (2025). Cardiac Rehabilitation Registries as Tools for Quality Improvement and Research: Insights From the SWEDEHEART-CR registry. Can. J. Cardiol..

[48] Sugiharto F., Nuraeni A., Trisyani Y., Melati Putri A., Aghnia Armansyah N. (2023). Barriers to Participation in Cardiac Rehabilitation Among Patients with Coronary Heart Disease After Reperfusion Therapy: A Scoping Review. Vasc. Health Risk Manag..

[49] Guo J., Huang P., Wu L., Meng X. (2026). Effects of Health Action Process Approach (HAPA)-Guided Exercise Rehabilitation on Cardiovascular Risk Factors in Perimenopausal Women with CHD: A Randomized Controlled Trial. Int. J. Women’s Health.

[50] Herber O.R., Smith K., White M., Jones M.C. (2017). ‘Just not for me’—Contributing factors to nonattendance/noncompletion at phase III cardiac rehabilitation in acute coronary syndrome patients: A qualitative enquiry. J. Clin. Nurs..

[51] Uddin J., Faruque M., Mashreky S.R., Siddiqueea Y., Chowdhury M.A., Karim R., Khaled M.F.I., Taylor L., Dalal H.M., Taylor R. (2026). Assessment of the Bangla Heart Manual in patients with coronary heart disease and their caregivers in Bangladesh: A feasibility study. BMJ Open.

[52] Desveaux L., Saragosa M., Russell K., McCleary N., Presseau J., Witteman H.O., Schwalm J.D., Ivers N.M. (2020). How and why a multifaceted intervention to improve adherence post-MI worked for some (and could work better for others): An outcome-driven qualitative process evaluation. BMJ Open.

[53] Ivers N.M., Schwalm J.D., Bouck Z., McCready T., Taljaard M., Grace S.L., Cunningham J., Bosiak B., Presseau J., Witteman H.O. (2020). Interventions supporting long term adherence and decreasing cardiovascular events after myocardial infarction (ISLAND): Pragmatic randomised controlled trial. BMJ.

[54] Fallah Z., Feizi A., Sadeghi M., Hadavi M.M., Shahnazi H. (2025). The effect of home-based virtual exercise rehabilitation on myocardial infarction patients using the health action process approach: A randomized controlled trial. BMC Sports Sci. Med. Rehabil..

[55] da Silva Vieira Á.S., de Melo M.C.D.A., Noites S.P.A.R.S., Machado J.P., Gabriel M.M.J. (2017). The effect of virtual reality on a home-based cardiac rehabilitation program on body composition, lipid profile and eating patterns: A randomized controlled trial. Eur. J. Integr. Med..

[56] Lee Y., Huang C.Y., Ho H., Cheng Y.Y. (2026). Effectiveness of Cardiopulmonary Exercise Testing as an Incentive to Enhance Outpatient Cardiac Rehabilitation Participation in Acute Coronary Syndrome Survivors: A Study Protocol for a Randomized Controlled Trial with Determinant Analysis. J. Clin. Med..

[57] Mishina I.E., Blinova K.A., Gudukhin A.A., Kopysheva E.N. (2026). Remote ECG-Guided Cardiac Rehabilitation: An Outpatient Model Using the Accordix Platform. Curr. Cardiol. Rev..

[58] Dasbiswas A., Shelke A., Singhal A., Shah B. (2020). Consensus statement: Improvement in care and outcomes for patients with ST-elevation myocardial infarction with timely reperfusion therapy: A pharmaco-invasive approach. Int. J. Adv. Med..

[59] Persiyanova-Dubrova A.L., Bubnova M.G., Aronov D.M., Matveeva I.F. (2024). Maintaining optimal physical activity of patients following cardiac rehabilitation. CardioSomatics.

[60] Kotseva K., De Backer G., De Bacquer D., Rydén L., Hoes A., Grobbee D., Maggioni A., Marques-Vidal P., Jennings C., Abreu A. (2019). Lifestyle and impact on cardiovascular risk factor control in coronary patients across 27 countries: Results from the European Society of Cardiology ESC-EORP EUROASPIRE V registry. Eur. J. Prev. Cardiol..

[61] Rawstorn J.C., Gant N., Direito A., Beckmann C., Maddison R. (2016). Telehealth exercise-based cardiac rehabilitation: A systematic review and meta-analysis. Heart.

[62] Tessitore E., Sigaud P., Meyer P., Mach F. (2017). Activité physique au long cours après un infarctus du myocarde: Un défi permanent [Long-term physical activity after a myocardial infarction: A permanent challenge]. Rev. Med. Suisse..

[63] Coppi F., Pagnoni G., Grossule F., Nassar A., Maini A., Masaracchia G., Sbarra F., Battigaglia E., Maggio E., Aschieri D. (2025). Gender-Specific Differences in Diastolic Dysfunction and HFpEF: Pathophysiology, Diagnosis, and Therapeutic Strategies. J. Cardiovasc. Dev. Dis..

[64] Pagnoni G., Bolognesi M.G., Bricoli S., Rossi L., Arata A., Aschieri D. (2026). Out-of-Hospital Cardiac Arrest: Public-Access Defibrillation and System Approaches to Minimize Avoidable Delay. J. Clin. Med..

[65] Aschieri D., Bricoli S., Rossi L., Ferraro S., Bolognesi M.G., Nani S., Pelizzoni V., Capucci A. (2025). Improved Survival With Automated External Defibrillator-Only Training in a Public-Access Defibrillation Program: A 23-Year Database Analysis of Progetto Vita. J. Am. Heart Assoc..

[66] Savonitto G., Barbisan D., Ameri P., Lombardi C.M., Driussi M., Gentile P., Howard L., Toma M., Pagnesi M., Collini V. (2025). Characteristics, Prognosis, and European Society of Cardiology and European Respiratory Society Risk Stratification in Patients With Obesity and Pulmonary Arterial Hypertension. Chest.

[67] Pagnoni G., Giuggioli D., Darroudi S., de Pinto M., Maini A., Spinella A., Sandri G., Pinti M., Aschieri D., Baccarani A. (2026). Reproducibility of Echocardiographic Measurements of Right Ventricular Function: Comparison Between TAPSE and TDI S’. J. Cardiovasc. Dev. Dis..

[68] de Pinto M., Coppi F., Spinella A., Pagnoni G., Morgante V., Macripò P., Boschini M., Guerra A.F., Tampieri F., Secchi O. (2024). The predictive role of the TAPSE/sPAP ratio for cardiovascular events and mortality in systemic sclerosis with pulmonary hypertension. Front. Cardiovasc. Med..

[69] Darroudi S., Ghazaee H., Babaei N., Hosseini Z.S., Jamili M.J., Ensan B., Donyadide G., Pagnoni G., Coppi F., Shahri H.H. (2026). The effect of empagliflozin on inflammation and oxidative stress in patients undergoing percutaneous coronary intervention, a randomized controlled trial. Sci. Rep..

